# Pelvic splenosis—a very unusual location

**DOI:** 10.1259/bjrcr.20160026

**Published:** 2017-04-06

**Authors:** Raquel Lameiras, António P Matos, Carlos Luz, João Palas, Miguel Ramalho

**Affiliations:** ^1^Department of Radiology, Hospital Garcia de Orta, Almada, Portugal; ^2^Department of Surgery, Hospital Garcia de Orta, Almada, Portugal

## Abstract

We present a rare case of pelvic splenosis, and its imaging findings, in a 54-year-old female complaining of pelvic pain and vaginal bleeding for several months. Splenosis is a benign acquired condition defined as heterotopic auto-transplantation of splenic tissue to other compartments of the body and occurs after rupture of the spleen, either traumatic or iatrogenic. Symptoms are unspecific and vary according to the location of the implants; commonly the presenting symptom is abdominal pain or enlarging abdominal mass. Owing to its intrinsic properties and safety, magnetic resonance imaging is a valuable imaging modality, in which the splenosis implants may be securely identified, showing the same signal characteristics and enhancing patterns of the normal spleen, especially visualization of serpiginous enhancement on the arterial phase is virtually diagnostic.

## Background

Splenosis is a benign acquired condition defined as heterotopic auto-transplantation of splenic tissue to other compartments of the body.^1,2^ It occurs after rupture of the spleen, either traumatic or iatrogenic. It is estimated that splenosis develop in up to 67% of splenic injuries.^1^ The main mechanism of this entity is the disruption of the splenic capsule, either from trauma or surgery, allowing direct dissemination of splenic tissue to other locations.^3^

Splenosis differs from accessory spleens and polysplenia, both congenital conditions, unrelated to splenectomy. Accessory spleens, usually fewer in number, are supplied by the splenic artery, in contrast to splenosis in which the implant’s blood supply derives from the nearby vessels.^1^ Symptoms are non-specific and vary according to the location of the implants.

Splenosis is usually an incident finding in ultrasound or computed tomography (CT), which cannot definitively determine a splenic origin for the masses, although on unenhanced and contrast-enhanced CT these masses have similar attenuation to the expected normal splenic tissue.^2^ Owing to the excellent soft tissue contrast resolution, MRI may be the imaging method of choice.

## Clinical presentation

A 54-year-old female was admitted to our institution owing to complaints of pelvic pain and vaginal bleeding for several months. There was a history of appendectomy and splenectomy, the latter owing to abdominal trauma in childhood. In the physical examination there was pelvic tenderness. The remaining physical examination was unremarkable.

## Investigations/imaging findings

Laboratory tests were within normal values, including tumour markers, including cancer antigen 125 (CA-125), cancer antigen 19-9 (CA 19-9), beta-human chorionic gonadotropin (β-HCG) and alpha-fetoprotein (AFP).

Endovaginal ultrasound revealed multiple uterine fibroids and heterogeneous adnexal solid masses, showing rich vascularity on colour Doppler examination.

**Figure 1.**
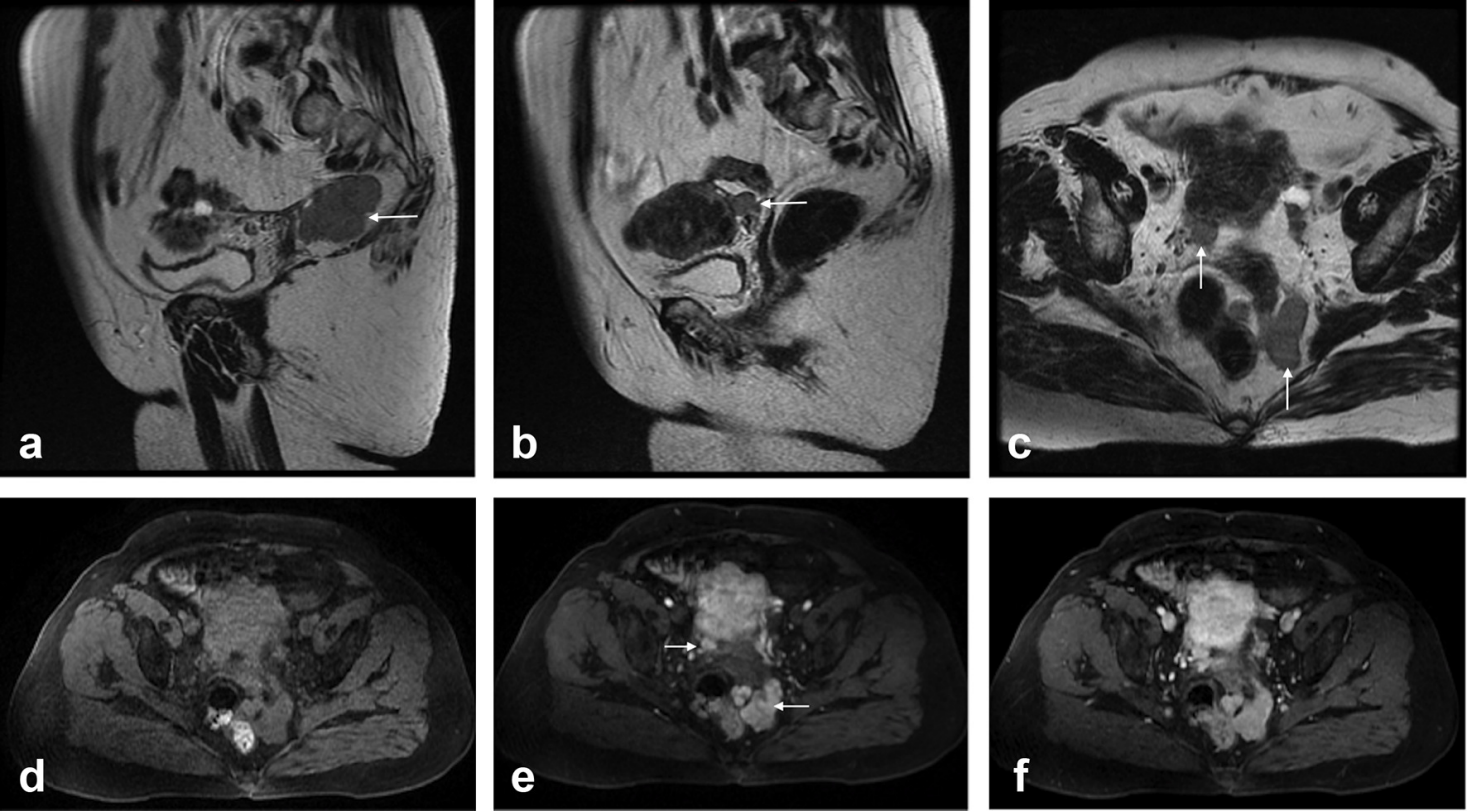
Sagittal (a,b) and transverse (c) fast spin-echo *T*_2_ weighted images. Transverse 3D gradient echo *T*_1_ weighted images pre- (d) and postcontrast images at the arterial (e) and venous (f) phases. Two homogeneous solid lesions are depicted in the pelvis, with mildly high signal intensity on *T*_2_ weighted images (arrows a–c). These lesions showed low signal on precontrast *T*_1_ weighted images (d). On the arterial phase (e), a strong and heterogeneous enhancement is shown. The largest lesion shows serpigenous enhancement (arrow, e). On the venous phase (f), the enhancement becomes homogeneous.

Gadolinium-enhanced magnetic resonance imaging (MRI) was performed for further characterization (Figure 1). The uterus revealed adenomyosis and myometrial solid lesions consistent with fibroids. Furthermore, two adnexal solid lesions were found. One well-defined nodule measuring 1 cm adjacent to the right ovary and to the right posterior wall of the uterine body. The other nodule measured 5.2 cm and was located near the posterior wall of the uterine cervix, left-sided in the recto-uterine pouch, with multilobulated contour. Both lesions showed homogeneous low *T*_1_ signal and homogeneous intermediate to high *T*_2_ signal. Both lesions were hypervascular on the arterial phase, showing strong and heterogeneous enhancement, the largest one showed serpiginous-like arterial enhancement, progressing to homogeneous signal intensity on venous and late phases images. On the upper abdomen, the native spleen was notably absent with multiple small left subphrenic solid nodules. These features were prospectively suggested of pelvic splenosis.

## Differential diagnosis

According to the clinical presentation and imaging findings, the differential diagnosis included pedunculated fibroids or adnexal lesions, including primary ovarian and tubarian tumours, and metastatic tumours. Primary ovarian neoplasms included (divided by histologic origin) epithelial tumours (most frequently serous and mucinous tumours), germ cell tumours (most frequently teratomas) and sex cord–stromal tumours (most frequently fibrothecoma).

Owing to the history of splenectomy, in the context of splenic trauma, pelvic splenosis was also included.

## Treatment

**Figure 2.**
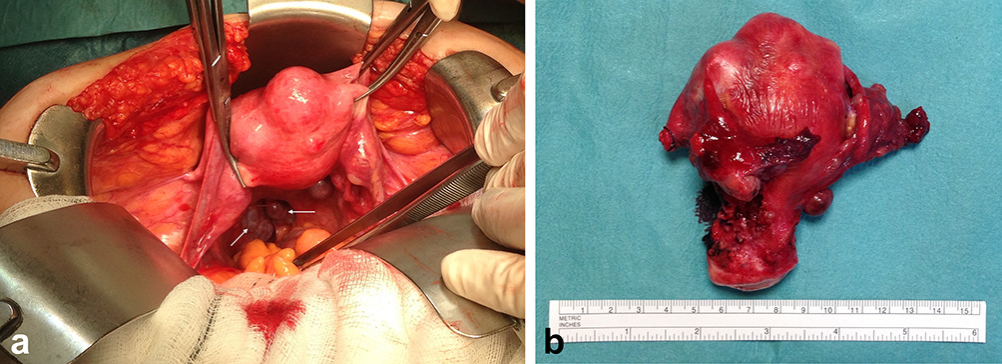
Images during surgery. Dark red to blue-black elastic lobulated nodule located on rectouterine pouch, visually suggestive of splenosis (arrow, a). Small nodule attached to uterine posterior wall suggestive of pelvic splenosis (surgical specimen is presented in b).

In order to treat the vaginal bleeding, hysterectomy with bilateral adnexectomy was performed through laparotomy (Figure 2). The largest lesion located on rectouterine pouch, was firmly attached to local tissues and was left untouched, as it was macroscopically suggestive of splenosis.

**Figure 3.**
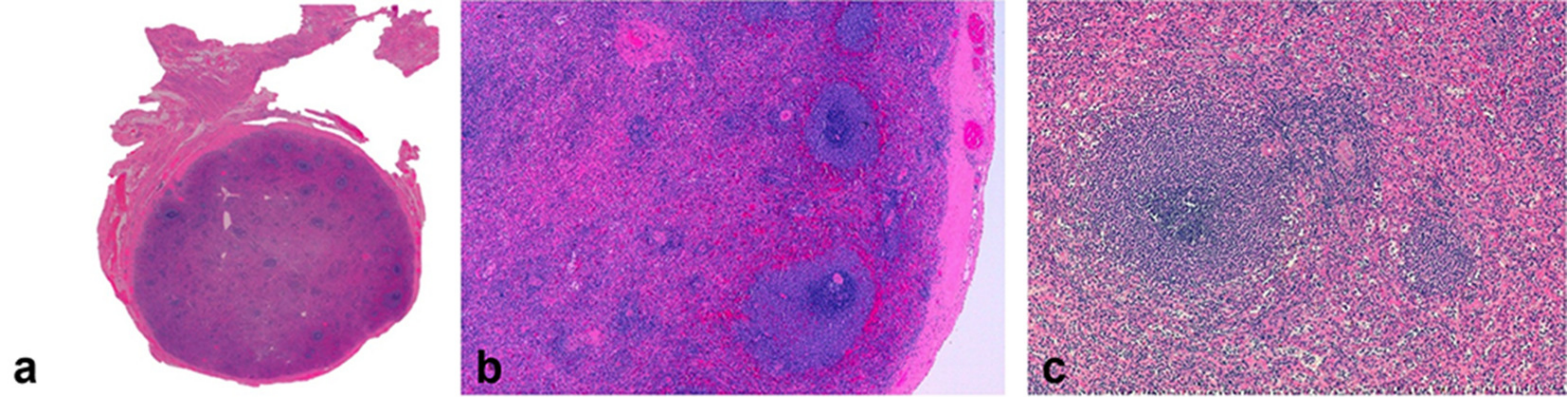
Histopathology (haematoxylin and eosin stain—a, no magnification; b, magnification ×40; c, magnification 100x). (a) There is a secondary follicle with the germinal center surrounded by the marginal zone and mantle zone while in the upper part of the image there is primary follicle without the germinal centre (b). Areas of lymphocytes clusters (but also neutrophils) that make up the white pulp, alternating with areas of red pulp (c).

Histopathological analysis of the nodule attached to the uterine body revealed thin and dense fibroelastic outer capsule, surrounding red and white pulp, showing central arteriole with lymphocytes areas. It was possible to see a primary and a secondary follicle containing a germinal centre (Figure 3). These findings were consistent with ectopic spleen. There was no evidence of malignancy.

## Outcome and follow-up

There were no surgical complications and after few days of recovery the patient was discharged asymptomatic. During follow-up (3 years) the patient remained asymptomatic.

## Discussion

Our patient presented with vaginal bleeding, pelvic pain and tenderness. We believe that these symptoms were primary related to the adenomyosis, while pelvic splenosis was an incidental finding. Supporting this assumption is the fact that symptoms resolved after surgery, even after leaving in place the largest splenosis implant. Pelvic splenosis commonly presents with pelvic pain or pelvic mass.^1^ However, patients may remain asymptomatic, being often incidentally diagnosed during unrelated surgery or imaging studies for other causes.^3^

On MRI splenosis implants have the same signal characteristics and enhancing patterns of the expected normal splenic tissue. On unenhanced sequences, splenosis implants show homogenous hypointensity on *T*_1_ weighted imaging (WI) and hyperintensity on *T*_2_ weighted images (in case of iron deposition they may show hypointensity on *T*_2_ weighted images).^4^ Similar to normal spleen, splenosis shows an arterial heterogeneous/ serpiginous enhancement pattern with later progressive homogenization during the dynamic studies;^4^ however, recognition of this arterial pattern may depend on the implant size, being easier to recognize in larger implants. This contrast enhancement pattern is also seen on dynamic CT imaging; however, MRI has the advantage of routine use of dynamic multiphasic acquisitions and lacking ionizing radiation.^2^ Furthermore, using superparamagnetic iron oxide (SPIO), a tissue-specific contrast agent for liver and spleen will allow the definitive diagnosis.^1,3^ SPIO is taken up by reticuloendothelial cells; therefore, splenosis demonstrates decreased signal on post-SPIO *T*_2_ weighted images compared with pre-SPIO *T*_2_ weighted images indicating the presence of splenic tissue.^5^ Therefore, SPIO imaging may be accepted as the reference test for the diagnosis of splenosis.

Although MRI with administration of SPIO is specific for diagnosis, the current diagnostic tool of choice is still scintigraphy with agents that distribute in the reticuloendothelial system such as Tc-99 m sulphur colloid, Tc-99 m heat-damaged erythrocytes or In-111 labelled platelets, allowing to detect small nodules missed on MRI.^1^

Pelvic splenosis in a woman can be a challenging diagnosis because of non-specific clinical presentation and the multiple gynaecological differentials.^6^ Previous case reports of pelvic splenosis have also emphasize this difficulty.^7,8^ Primary ovarian tumours with epithelial origin are the most frequent ovarian neoplasms, accounting for 60% of all neoplasms and 85% of malignant ovarian neoplasms.^9^ Thus, the most frequent epithelial tumours, cystadenoma and cystadenocarcinoma, should be considered. Distinctive to our case, these neoplasms are cystic lesion, frequently with large dimensions and they show areas of very high signal intensity on *T*_2_ weighted images, and thickened progressive enhancing septations and papillary projections. Furthermore, cystadenocarcinoma may show ascites, peritoneal implants, direct locoregional invasion and elevation of tumour markers (specifically, CA-125). Teratomas are by far the most frequent germ-cell tumour. Mature teratoma is a benign lesion, showing a broad spectrum of findings, ranging from purely cystic, with very high signal intensity on *T*_2_ weighted images and no enhancement on post-gadolinium MRI sequences, to heterogeneous or non-cystic mass, the latter composed predominantly of fat, showing high signal intensity on *T*_1_ weighted images and drop of signal on fat-suppressed MRI sequences. None of these features was seen on our case. Regarding sex-cell tumours, fibrothecoma are the most frequent lesions. Fibrothecomas, techomas and fibromas tend to share similar features including hypointensity on *T*_2_ weighted images and very little enhancement on early phases, with variable progressive enhancement. On the other hand endometriomas show very high signal intensity on unenhanced *T*_1_ weighted images, shading on *T*_2_ weighted images and little to no appreciable enhancement after gadolinium injection. Furthermore the characteristics of the pelvic nodules in our case were not specific of germ-cell and sex-cell tumours, the tumour markers associated with these entities were also negative.

Pedunculated fibroids are expected to have similar heterogeneous signal and enhancement as other fibroids. Moreover, the bridging vascular sign (vessels and/or signal voids that extend from the uterus to supply a pelvic mass),^10^ characteristic of pedunculated fibroids, was not present.

Metastases are not rare to the adnexa, usually from the endometrium, breast, colon, stomach and cervix primary tumours. Our patient had no history of neoplastic disease.

Primary fallopian tube carcinoma is very rare, accounting for approximately 0.14–1.8% of female genital malignancies.^11^ However, the prevalence may be underestimated because it is difficult to differentiate it from epithelial ovarian carcinoma.^9^ Imaging findings vary, and may appear as a fluid-filled tubular adnexal structure containing nodular or papillary solid components or as a multilocular cystic mass. On MRI the solid component is usually hypointense on *T*_1_ weighted images and iso to hyperintense on *T*_2_ weighted images.^12^ There are also associated findings such as intrauterine fluid collection, peritumoural ascites and hydrosalpinx.^11,12^ The appearance of the pelvic nodules in our case was not suggestive, ancillary findings were absent and tumour markers were negative.

In this case the diagnosis of pelvic splenosis was suspected based on imaging characteristics of the nodules on MRI, especially the hypervascular and serpiginous enhancement seen in the largest lesion, which was confirmed by histopathological analysis of the resected nodule. Pelvic splenosis is a benign disease and in asymptomatic cases usually there is no need for further treatment.^6^ Nevertheless, surgery is the ideal treatment of choice in symptomatic cases, while minimally invasive surgery such as laparoscopy may be the ideal treatment.^6^

Splenosis may also occur in other locations in the abdomen and may mimic, e.g. liver or colon tumours.^11,12^ Our case emphasizes how challenging a diagnosis of pelvic splenosis can be, especially in a woman as there are various differential diagnoses to take into consideration.

## Conclusions

In conclusion, pelvic splenosis is a rare benign condition whose radiological diagnosis is difficult on US, CT and routine MRI without the history of trauma (blunt or penetrating) of the spleen. Despite the number of differential diagnosis it is important that radiologists be alert for this diagnosis and recognize the MR imaging characteristics of splenosis as it may allow the definite diagnosis. Splenosis show an arterial heterogeneous/serpiginous enhancement pattern with later progressive homogenization during the dynamic studies; however, recognition of this arterial pattern may depend on the implant size, making it easier to recognize in larger ones. For the definitive diagnosis of small ones, routine MRI may not always be a useful modality.

## Learning points

1. MRI is useful detection and characterization of splenosis.
2. Splenosis implants show similar signal intensity and enhancement to those of a spleen in all sequences.
3. Identification of serpiginous enhancement on the arterial phase is virtually diagnostic, but it’s not seen in all splenosis cases, especially in small sized ones, so the diagnosis may be sometimes challenging.

## Consent

Informed consent to publish this case (including images and data) was obtained and is held on record.

## References

[1] Tsitouridis I, Michaelides M, Sotiriadis C, Arvaniti M. CT and MRI of intraperitoneal splenosis. Diagn Interv Radiol 2010; 16: 145–9. 19838993 10.4261/1305-3825.DIR.1855-08.1

[2] Lake ST, Johnson PT, Kawamoto S, Hruban RH, Fishman EK CT of splenosis: patterns and pitfalls. Am J Roentgenol 2012; 199: 686–93. 10.2214/AJR.11.7896 23169741

[3] Storm BL, Abbitt PL, Allen DA, Ros PR. Splenosis: superparamagnetic iron oxide-enhanced MR imaging. Am J Roentgenol 1992; 159: 333–5. 1632350 10.2214/ajr.159.2.1632350

[4] Lin WC, Lee RC, Chiang JH, Wei CJ, Chu LS, Liu RS, et al MR features of abdominal splenosis. Am J Roentgenol 2003; 180: 493–6. 12540458 10.2214/ajr.180.2.1800493

[5] Herédia V, Altun E, Bilaj F, Ramalho M, Hyslop BW, Semelka RC. Gadolinium- and superparamagnetic-iron-oxide-enhanced MR findings of intrapancreatic accessory spleen in five patients. Magn Reson Imaging 2008; 26: 1273–8. 18440173 10.1016/j.mri.2008.02.008

[6] Park SY, Kim JY, Lee JH, Choi JS, Ko JH, Park SH. Laparoscopic management of pelvic splenosis. Obstet Gynecol Sci 2014; 57: 89–91. 24596826 10.5468/ogs.2014.57.1.89 PMC3924747

[7] Segev Y, Lavie O, Goldberg Y, Kaufman Y, Peerl G, Gips S, et al Pelvic splenosis mimicking an ovarian mass: a non-invasive approach. Isr Med Assoc J 2007; 9: 819–20. 18085043

[8] Metindir J, Mersin HH, Zarife Bulut M. Pelvic splenosis mimicking ovarian metastasis of breast carcinoma: a case report. J Turk Ger Gynecol Assoc 2011; 12: 130–2. 24591977 10.5152/jtgga.2011.30 PMC3939106

[9] Jung SE, Lee JM, Rha SE, Byun JY, Jung JI, Hahn ST. CT and MR imaging of ovarian tumors with emphasis on differential diagnosis. Radiographics 2002; 22: 1305–25. 12432104 10.1148/rg.226025033

[10] Kim JC, Kim SS, Park JY. “Bridging vascular sign” in the MR diagnosis of exophytic uterine leiomyoma. J Comput Assist Tomogr 2000; 24: 57–60. 10667660 10.1097/00004728-200001000-00012

[11] Pectasides D, Pectasides E, Economopoulos T. Fallopian tube carcinoma: a review. The Oncologist 2006; 11: 902–12. 16951394 10.1634/theoncologist.11-8-902

[12] Rezvani M, Shaaban AM. Fallopian tube disease in the nonpregnant patient. Radiographics 2011; 31: 527–48. 21415195 10.1148/rg.312105090

